# Changes in mineral forms of nitrogen and sulfur and enzymatic activities during composting of lignocellulosic waste and chicken feathers

**DOI:** 10.1007/s11356-019-04453-2

**Published:** 2019-02-13

**Authors:** Justyna Bohacz

**Affiliations:** Faculty of Agrobioengineering, Department of Environmental Microbiology, Laboratory of Mycology, University of Life Sciences in Lublin, 7 Leszczyńskiego Street, 20-069 Lublin, Poland

**Keywords:** Composting, Lignocellulosic waste, Compost water extracts, Sulfates, Chicken feathers

## Abstract

The aim of this study was to show the dynamics of changes in the activity of enzymes responsible for C, N, and S metabolism, i.e., cellulase, protease, urease, and arylsulfatase in two lignocellulosic composts as well as changes in the concentration of mineral forms important in plant nutrition (N-NH_4_^+^, N-NO_3_^−^, S-SO_4_^2−^). Most of the enzyme activity was higher during 10 weeks of composting in compost I, containing higher amounts of easily available organic matter than in compost II. Enzymatic activities in compost II remained at a higher level for a longer time, but they increased at a slower rate. Mineral content changes in the compost mass consisted primarily of an increase in N-NO_3_^−^ concentration and a decrease in N-NH_4_^+^ and S-SO_4_^2−^ levels, especially in compost I. The concentration of mineral nitrogen and sulfur forms in compost water extracts was about 10–100 times lower than in the compost mass. At the end of composting, the amount of sulfates in the compost mass was 30 and 150 mg kg^−1^ dw in compost II and I, respectively. In this context, the composts obtained should be considered valuable for fertilizing soils poor in this component and for cultivating plants with high sulfate S demand.

## Introduction

Lignocellulosic waste is often composted or co-composted. It is mainly produced by mechanical processing of wood and includes branches, sawdust, and bark. According to Sánchez (2009), global annual production of this waste ranges from 0.077 to 380 tons × 10^6^/year. Szwed and Bohacz (2014) reported that over 2.5 million Mg of organic waste from the wood processing industry and 9.0 million Mg of agricultural waste are generated in Poland. Lignocellulosic wastes undergo slow biotransformation and biodegradation due to their hard-to-degrade nature, resulting from the chemical structure (Sánchez 2009). However, such wastes are the main precursors of humus along with other microbiological biodegradation products. The quality of the final product of the composting process is conditioned by the activity of different groups of microorganisms dictated by the chemical composition of the composted materials, and it is evaluated on the basis of biological, chemical, and physical parameters.

Microbiological activity determines the stability and maturity of composts; it is expressed in microbial biomass formation and the number of mesophilic and thermophilic bacteria, oxygen uptake, and CO_2_ release as well as changes in enzymatic activity in the composted mass (Barrena et al. 2008; Cunha-Queda et al. 2007; Jurado et al. 2014). Studies of different authors have shown that microbial biomass, particularly of thermophilic bacteria, decreases as the compost stabilizes and reaches maturity, as does the oxygen uptake and CO_2_ emission. Microorganisms capable of degrading polymers in the composted mass produce a complex of extracellular enzymes (Jurado et al. 2014). The dynamics of changes of some enzymatic activities is recognized by many authors as biological indicators of compost maturity (Bohacz and Korniłłowicz-Kowalska 2009a; Castaldi et al. 2008). This is due to the fact that biochemical degradation of organic matter in the composted mass is catalyzed by specific hydrolytic enzymes, including cellulases that depolymerize cellulose, β-glucosidases that hydrolyze glucosides, aminohydrolases, proteases, and ureases involved in organic N mineralization, phosphates that hydrolyze organic phosphorus compounds into inorganic forms and arylsulfatases that produce sulfates from organic sulfur compounds of the composted mass and also lipases (Cunha-Queda et al. 2007; Vargas-García et al. 2010). Barrena et al. (2008) also mentioned respiratory activity and dehydrogenase activity as indicators of microbiological activity of the composting process. Bohacz (2017) described the dynamics of changes in ligninolytic enzyme activities, such as ligninases (LiP), HR peroxidase (HRP), and ligninolysis auxiliary enzymes, such as glucose oxidase (GOD) in successive biothermal phases in composts containing pine bark, sawdust, grass, straw, and chicken feathers.

Enzymatic transformation of organic matter that occurs during composting is caused by microorganisms. Metabolism products found in the water-soluble phase are not only the source of C, N, and energy for successively occurring microorganisms, but also provide information on the advancement of the composting process (Said-Pullicino et al. 2007). Shrestha et al. (2012) have reported that compost extracts, commonly called “compost tea,” are the source of nutrients for plants and also argued that compost teas could have a potentially positive effect on plants. This fraction contains soluble polysaccharides and proteins, organic polymer decomposition products, enzymes, and repolymerized and mineral compounds, whose concentrations determine application conditions of these composts to the soil and their use in foliar nutrition of plants. El-Gohary et al. (2010) reported that the mixture of humic acids with potassium, phosphorus, calcium, iron, and sulfur salts can be quickly absorbed by plants in the soil or as foliar applications.

Therefore, the evaluation of compost utility value should include its water extracts, which until now have rarely been analyzed in composting studies and rarely used in practice, e.g., in horticulture for foliar plant feeding. The main purpose of the present work was to evaluate the compost mass in terms of biochemical and chemical parameters, and especially water extracts of composts obtained from lignocellulose and feather waste based on chemical parameters, particularly those concerning mineral forms of N and S. A novel aspect of the study was the evaluation of compost extracts as a potential preparation for foliar plant nutrition.

## Materials and methods

### Materials

Composts containing lignocellulosic waste were prepared in two variants, each with a ratio of C/*N* = 25. Concentrations of C and N in the input material are given in the article of Bohacz (2018b). The first composting variant (compost I- PGSF) contained 42.86% pine bark, 34.28% grass, 20.00% sawdust, and 2.86% broiler chicken feathers. The second composting variant (compost II-PSSF) contained 25.54% pine bark, 10.63% wheat straw, 51.07% sawdust, and 12.76% broiler chicken feathers. Composting was carried out in 32-L containers with perforated bottoms. Containers were wrapped with foil filled with styrofoam to prevent temperature loss. The composted mass was moistened to approximately 60% of the total water capacity. Composts were mixed to maintain aeration twice a month during 14 weeks and after that time once a month (Bohacz 2018a). The temperature of the composted mass was monitored using a mercury thermometer, which was present all the time in the compost mixture. The description of temperature changes during composting of lignocellulosic waste (compost I and II) is presented in the study of Bohacz (2018a). The highest temperatures were measured on day 12 of composting (44 °C) and on day 4 of composting (40 °C) in compost I (PGSF) and compost II (PSSF), respectively. The criterion for selecting composted materials concerned not only the scale of their formation, but also eliminating the risk of nitrate leaching by composting wood shavings, as indicated by Tognetti et al. (2007), and the possibility of improving the structure and abundance of minerals in the soil fertilized with composts.

### Enzymatic analysis of compost solid fractions

Biochemical analyses in the composted mass were carried out at time point 0, 18 h after starting the composting and after 2, 4, 8, 10, 14, 18, 22, 26, and 30 weeks of composting, which was related to the division into biothermal phases based on temperature changes in the composted mass. The division into biothermal phases is described in the study of Bohacz (2018a). The analyses included determination of protease (PA), urease (UA), and cellulase (CA) activities. Protease activity was determined in 2-g compost samples using casein as a substrate, incubated in 0.1 M Tris-HCl buffer pH 8.1 for 1 h, at 50 °C (Ladd and Butler 1972). Urease activity was determined in 5-g of compost samples using urea solution as substrate, incubated for 18 h, at 37 °C (Zantua and Bremner 1975), with some modifications as suggested by Furczak et al. (1991). Cellulase (endo-glucanase) activity was measured according to Pancholy and Rice (1973) with carboxymethylcellulose (CMC) as a substrate. The reaction was carried out in 0.1 M acetate buffer pH 5.3, at 30 °C, for 24 h. Arylsulfatase activity (AA) was measured as described in the study of Alef and Nannipieri (1995) in 0.5 M acetate buffer pH 5.8 using p-nitrophenyl sulfate as a substrate for 1 h, at 37 °C.

### Gas volatilization determination

Volatilization of gaseous ammonia was determined by the method of Kim (1973). Gaseous H_2_S volatilization was also determined due to the presence of a large amount of sulfuric amino acid (cystine) in chicken feathers. The presence of H_2_S in the composting environment was analyzed by placing a strip of filter paper soaked with lead acetate in a tube (under the plug). The released H_2_S reacted with this compound to produce lead sulfide, which darkened the strip. The presence of black precipitate was evidence of desulfuration.

### Water extract preparation

A representative sample (100 g) of the compost mass was collected from several places of the surface and middle layer on successive experimental dates, i.e., after mixing the materials used for composting (time point 0) and after 14 and 30 weeks. The sample was suspended in 500 mL distilled water and shaken for 1 h on a rotary shaker; filtered and distilled water was added to a final volume of 1000 mL.

### Analysis of mineral forms of N

Chemical analyses in compost mass and water extracts from composts included N-NO_3_^−^ determination by ion chromatography after 18 h (time point 0), and 14 and 30 weeks, and N-NH_4_^+^ concentration using flow spectrometry (compost water extracts) and distillation method (composts mass).

### Analysis of mineral form of S

Periodical analyses of mineral S form (S-SO_4_^2−^) in the compost mass and compost water extracts were carried out using ion chromatography.

All the above-mentioned chemical analyses in composts and in compost water extracts were carried out at the Main Chemical Laboratory, Institute of Soil Science and Plant Cultivation, Puławy, Poland.

### Statistical evaluation

Statistical analyses of the data were conducted using the STATISTICA 12 software. One-way analysis of variance (ANOVA) and multiple comparisons (Fisher’s test) were performed to compare statistically homogeneous groups for the tested means in individual biothermal phases. The *r* Pearson correlation analysis was performed in order to demonstrate the correlation between enzyme activity and the concentration of mineral nitrogen forms, at the significance level of *α* = 0.05.

## Results and discussion

The use of various maturity and stability indicators of composts provided a complete picture not only of the transformation of organic matter in the composting of organic waste, but also helped to assess the utility value of these composts.

### Compost enzymatic activities

Bohacz and Korniłłowicz-Kowalska (2009a) and Usmani et al. (2018) showed that hydrolytic enzymes, due to their inductive nature, are good indicators of quantitative and qualitative changes in the content of individual organic polymers in the composting process. Additionally, Szwed and Bohacz (2014) reported that enzyme activities, as an indicator of organic matter metabolism in soil fertilized with compost, correlated significantly with soil chemical parameters. In addition, enzymatic activity profiles vary depending on the stage of composting (Tiquia 2002).

**Fig. 1:**
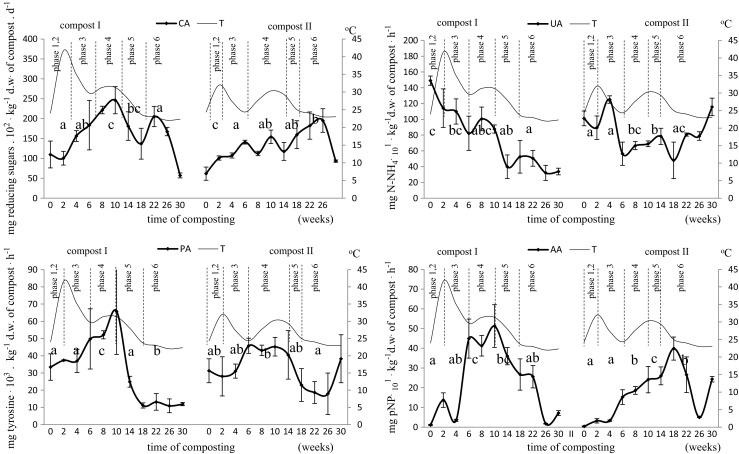
Changes in enzymatic activity during the composting of lignocellulosic and keratin waste. Statistically significant differences between phases are marked with different letters (*ɑ* = 0.05); means of three replicates ± standard deviation; the division into biothermal phases is described in the study of Bohacz (2018a): compost I (PGSF): phases 1 and 2 (weeks 0–2), phase 3 (weeks 2–6), phase 4 (weeks 6–10), phase 5 (weeks 10–18), phase 6 (weeks 18–30); compost II (PSSF) phases 1 and 2 (weeks 0–2), phase 3 (weeks 2–6), phase 4 (weeks 6–10), phase 5 (weeks 10–14), phase 6 (weeks 14–30); *T* temperature

The experiments conducted in this study showed that the distribution of organic matter in both compost variants was characterized by different biochemical activity (Fig. 1). Hydrolysis of polysaccharides, such as cellulose and proteins began after the mixing of composted materials. This was demonstrated by the increase in the activity of endoglucanase-type cellulase (CA), which breaks down the bonds within cellulose molecules, and protease (PA), which hydrolyzes protein peptide bonds. Endoglucanase activity (CA) increased faster in the early composting phases (phases 1 and 2) in the grass-containing variant (PGSF), which could be explained by better availability of this polysaccharide. Jurado et al. (2014) observed a similar dynamics of cellulase activity changes during composting of horticultural waste with pine chips. These authors recorded the highest activity of this enzyme after 2 and 42 days of composting. Endoglucanase-type cellulase activity in compost II (PSSF), containing less accessible lignocellulose fraction, was significantly higher only at the end of the composting period (phases 5 and 6). This effect coincided with a significant increase in ligninase activity, as described in my earlier work (Bohacz 2017). The convergence of maxima of cellulo- and ligninolytic activities in compost II indicated progressive degradation of the ligninocellulose complex.

Adopting protease activity as an indicator of the activity of enzymes hydrolyzing peptide bonds in composts (Bohacz and Korniłłowicz-Kowalska 2009a), it was found that the activity of these enzymes was increasing till the 10th week of composting both in composts I (PGSF) and II (PSSF). The current study demonstrated that intensive decomposition of the protein fraction of organic matter of both composts in the first 2 months of composting should be linked to microbial biomass synthesis. Choińska-Pulit et al. (2019) recorded an increase in protease activity during composting of pigs’ bristles with sawdust and lignite dust also during the first 2 weeks of composting followed by a decrease in the activity of this enzyme. Bohacz and Korniłłowicz-Kowalska (2009a) explained lower protease activity during the composting process by the inhibitory effect of simple carbon complexes released during polysaccharide hydrolysis. However, as shown in the present work (Fig. 1), the activity of this enzyme in a compost containing more difficult to degrade lignocellulose (compost II) was lower during the first 14 weeks of composting (to phase 4) despite a higher percentage of feather proteins. It was only in the 5th and 6th biothermal phase when protease activity was higher than in compost I. It should be assumed that the reason for this phenomenon was the low initial content of the easily available lignocellulose fraction in compost II, which provided organic C for the synthesis of microbial biomass (grass was the source of this fraction in compost I). The level of simpler carbon sources in compost II increased only in phases 5 and 6, as evidenced by the increase in endoglucanase activity, an enzyme releasing soluble sources of organic C from cellulose of the bark and sawdust lignocellulose complex. Thus, the nitrogen demand during this period was also higher in comparison to compost I, hence the higher protease activity at the end of phase 6 as well as an increase in N-NH_4_^+^ release till 14 weeks of composting (Fig. 1).

Urease, as an enzyme-releasing ammonium nitrogen, is involved in the final stage of degradation of organic nitrogen compounds. Urease can be synthesized under urea conditions or other alternative sources of N (Adetunji et al. 2017). High urease activity was noted especially in compost I in biothermal phases 1 and 2, which was likely associated with the use of N-NH_4_^+^ for microbial biomass synthesis, as mentioned above. After this time, the activity of this enzyme tended to decrease (Fig. 1). A similar profile of urease activity was described by Jurado et al. (2014) for urease activity in composts containing horticultural waste with pine chips. In compost II, urease activity varied and increased slightly in biothermal phase 3 and at the end of the process, which was coupled with the intensification of polysaccharide decomposition, i.e., cellulose. The decline in urease activity in compost I and II was most likely due to the depletion of available nitrogen compounds, and consequently, growth limitation of proteolytic microorganisms. This observation was in line with the view of Castaldi et al. (2008), who explained the decline in urease activity during composting by biomass reduction. The results obtained in this work pointed to yet another important factor causing lower urease activity. The high concentration of ammonium ion was inhibiting this enzyme biosynthesis. Therefore, the reduction of ammonium ion accumulation in the studied composts could have increased the activity of this enzyme in biothermal phase 6 in both composts I and II. Degradation of the protein component was slower in compost II (PSSF), containing more chicken feathers (12.76%) and the hard-degradable fraction of lignocellulose.

Arylsulfatase was determined due to the large amounts of organic sulfur in the keratin waste in the composted materials. This enzyme plays an important role in the process of organic sulfur mineralization, and thus making it available to plants in the form of sulfates. It was shown that the activity of this enzyme increased till the end of biothermal phase 4 in compost I with a lower organic S content (2.86% feathers). The activity of this enzyme in compost II (PSSF), containing over four times more of that component, increased slowly reaching maximum in biothermal phase 6, after which it decreased rapidly (Fig. 1). Mondini et al. (2004) obtained similar dynamics of changes in arylsulfatase activity in composts containing cotton wastes and yard wastes (grass, leaves, and prunings). The dynamics of changes in the activity of this enzyme was similar to protease activity in the composts tested, especially in compost I (PGSF). This would suggest a parallel course of proteolysis and mineralization of feather organic S during the composting process. This suggestion was consistent with the results of Korniłłowicz-Kowalska (1997), who indicated that sulfate release during microbial decomposition of native keratin feathers was correlated with the release and accumulation of peptides in the medium.

Most of the enzymatic activities tested were higher in compost I with grass, richer in easily accessible organic C and persisted till week 10 of composting. In compost II, which contained more difficult to assimilate organic C, and thus the decomposition was slower, enzymatic activities persisted at a higher level, but increased at a slower rate (Fig. 1).

### Changes in mineral N forms in the compost mass and compost water extracts

**Fig. 2:**
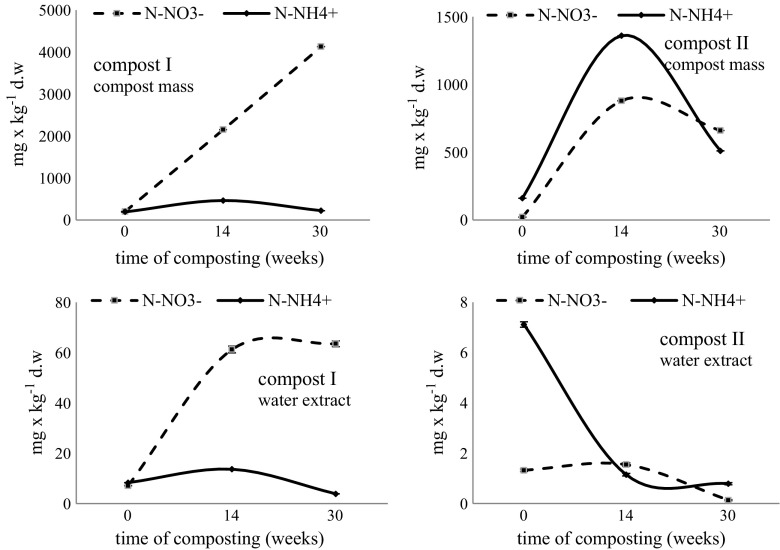
Changes in mineral form of N (mg kg^−1^ dw) in the compost mass and water extract of composts. Means of three replicates ± standard deviation

**Fig. 3:**
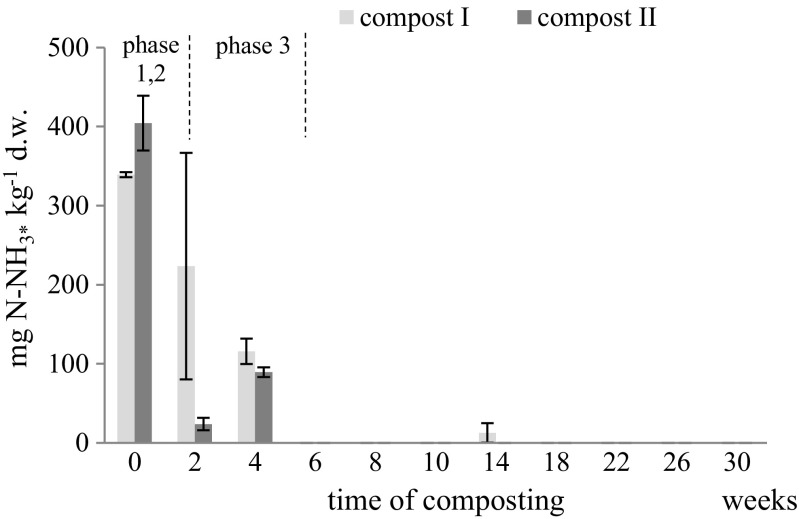
Dynamics of ammonia volatilization during composting of lignocellulosic and keratin waste. Means of three replicates ± standard deviation. Explanation as in Fig. 1

**Fig. 4:**
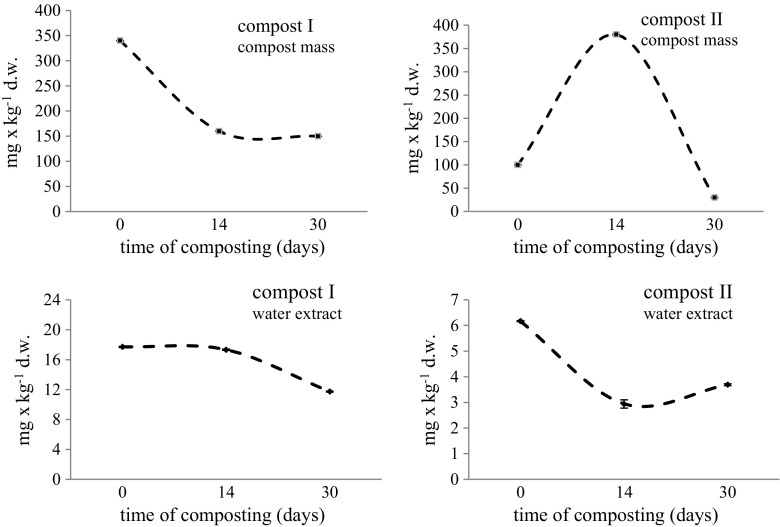
Changes in mineral forms of S (sulfates) in the compost mass and water extracts of composts. Means of three replicates ± standard deviation

Compost fertilizer value is defined by the concentration of macro- and micronutrients during composting, especially in the maturation and stabilization phases. Introducing mature composts to the soil is important not only from the point of view of enrichment in humus, but also preservation or improvement of plant health (Iwegbue et al. 2006; Shrestha et al. 2012; Wong 1985). In this work, it was found that changes in the enzymatic activity of microorganisms occurring on composting organic materials were accompanied by chemical changes in the composting mass and compost water extracts (Figs. 2, 3, and 4).

Mineral content changes in the compost mass (Fig. 2) consisted primarily of an increase in N-NO_3_^−^ concentration and a decrease in N-NH_4_^+^ and a parallel decrease of total N (especially in compost I), which was partly reported by the author in the work of Bohacz (2018b). A similar trend of N-NH_4_^+^ and N-NO_3_^−^ changes was observed during composting of pig manure and bulking agent, as described by Wu et al. (2017). Tognetti et al. (2007) argued that N-NH_4_^+^ concentration at the level of < 400 mg/kg indicated compost maturity. In compost II (PSSF), the values were higher in week 30 and amounted to 510 mg/kg, while they were lower in compost I (PGSF) and amounted to 220 mg/kg. A reduction in ammonium ion concentration during composting is the result of nitrification, ammonia volatilization, and immobilization by microorganisms during decomposition of organic matter (Raj and Antil 2011). Varma and Kalamdhad (2015) indicated that the decrease in N-NH_4_^+^ during composting of vegetable waste was associated with an increase in ammonia volatilization, especially at high temperature and pH increases.

On the other hand, as shown by Bohacz and Korniłłowicz-Kowalska (2009b), microorganism nitrification activity of both bacteria and fungi led to N-NO_3_^−^ increase at the end of composting. In compost II, similar dynamics of changes in N-NH_4_^+^ and N-NO_3_^−^ content till week 14 of composting indicated a progressive nitrification process. After this time, a decline of both forms of nitrogen was noted with the predominance of the nitrate form. Tognetti et al. (2007) reported that the reduction of nitrate values in compost with wood shavings and municipal solid waste was due to the increase in the total organic carbon/total nitrogen ratio (TOC/TN), which caused the immobilization of nitrogen by microorganisms. Brewer and Sullivan (2003) explained the decrease of nitrate ion content by leaching from the composted mass or intensified denitrification processes. At the end of composting in this study, the content of nitrate ions was higher than of ammonium ions (Fig. 2).

Assuming that changes in the content of different forms of nitrogen in the compost mass were not sufficient to evaluate the course of the composting process, these components were also determined in compost water extracts. Bohacz and Korniłłowicz-Kowalska (2009b) showed that this allowed to evaluate the bioavailability of these elements, but also the degree of compost maturation. In addition, as demonstrated by El-Gohary et al. (2010), compost extracts contain relatively higher amounts of organic matter, plant nutrients, and mineral salts than compost mass. In addition, the use of these extracts for foliar nutrition (spraying) also reduces the use of fertilizers and chemical plant protection agents and prevents plant nutritional deficiencies. El-Gohary et al. (2010) reported that aqueous solutions containing humic acids along with potassium, phosphorus, calcium, iron, and sulfur salts can be quickly absorbed by plants upon introduction into the soil as well as by foliar application. Ammonium nitrogen level in water extracts of the tested lignocellulosic composts was not high. Said-Pullicino et al. (2007) obtained lower ammonium nitrogen values in compost tea from municipal solid waste composting, yard trimmings, and tobacco residues. Ezz El-Din and Hendawy (2010) showed that spraying of compost tea with nitrogen content at the level of 249 ppm improved plant growth, increased fresh weight, and number of suckers and seed weight of *Borago officinalis*. The highest mean values of ammonium nitrogen in the extracts were found in compost I (PGSF) (50.33 mg kg^−1^ dw). An increase in the amount of N-NH_4_^+^ was observed at week 14 of the compost mass processing in compost I and a decrease in N-NH_4_^+^ concentration in compost II (Fig. 2). Overall, it was found that the amount of leachable ammonium nitrogen accumulated during composting was lower in comparison with the baseline level. This effect was probably associated with biological sorption in microbial cells and N-NH_4_^+^ nitrification. The increase in N-nitrate concentration in water extracts of both composts was significant at week 14 of composting (Fig. 2). The quantity of nitrate N during this period was higher in compost I than compost II. The content of these ions was higher at the end of the composting process compared to ammonium ions. Choińska-Pulit et al. (2019) showed that inoculating composted swine bristles together with sawdust and lignite dust with *B. cereus* PCM2849 led to increased release of water-soluble N-NO_3_ compared to non-inoculated composts. However, El-Haddad et al. (2014) obtained lower N-NO_3_^−^ values in compost tea from composted plant and animal waste.

### Ammonia volatilization

As a result of microorganism ammonification activity, nitrogen metabolism was also associated with ammonia volatilization, which occurred at the beginning of the composting process and lasted until week 4 (Fig. 3). Similar results were obtained during composting of sewage sludge (Li et al. 2018) and agro-food wastes (Santos et al. 2018). The intensity of ammonia volatilization was determined based on the composition of the composted mass. In compost I, containing less feathers grass, nitrogen losses in the form of gaseous ammonia ranged from 1.0 to 2.45% of organic nitrogen, while in compost II, with higher amount of feathers, from 0.2 to 4.0%. Nitrogen losses in the form of ammonia gas were in total the largest in the compost with lower amount of chicken feathers and grass addition and persisted to biothermal phase 3, reaching a maximum after 2 weeks. This phenomenon demonstrated intensive ammonification of feather proteins in this compost despite their four–five times lower content compared to compost II (2.86% and 12.76%, respectively). The effect observed in compost I indicated a high microbial population demand for nitrogen due to the higher content of readily available organic C (grass). In compost II, with higher feather content, increased ammonia emission was only observed in the initial composting phase (phase 1–2), when there were certain amounts of simple carbon sources available in the composted waste, contributing to the intensive growth of microorganisms, and thus the biological sorption of ammonium N. Oxygenation of the composted mass, pH, and temperature also influenced the volatilization of non-bound nitrogen in the cells. The correlation of ammonia volatilization with temperature increase of composts was demonstrated in the work by Pagans et al. (2006). The correlation (*α* = 0.05) between ammonia volatilization and temperature, at the level of 0.729 and 0.761*** in the PGSF and PSSF, respectively, was also shown in the present study. On the other hand, it is known that the temperature increase inhibits the nitrification process, and thus oxidation of N-NH_4_^+^ to N-NO_3_^−^. Ammonia volatilization was intensified under alkaline conditions of the composted biomass throughout three biothermal phases (pH about 7.0 in compost II and about 6.50 in compost II). Increasing pH of the compost mass resulted in the accumulation of released ammonium ions. Bazrafshan et al. (2016) and Waqas et al. (2018) reported the influence of pH on ammonia release from composts obtained from municipal solid wastes and food waste, respectively. These authors explained the volatilization of ammonia and the increase in pH by proton consumption, CO_2_ generation and organic nitrogen mineralization.

### Sulfate content in the compost mass and compost water extracts

High content of sulfuric amino acids in keratin proteins has also led to the analysis of microbiological transformation products of organic sulfur in the composting of lignocellulosic waste with keratin waste. The study of Bohacz and Korniłłowicz-Kowalska (2009b) demonstrated that sulfate content in the compost mass is a good indicator of the maturity of composts composed of feathers and lignocellulosic material. There is little data on the presence of sulfates in compost water extracts when fertilizers are considered. In addition to nitrogen, sulfur is one of the most important biogenic elements involved in plant metabolism, i.a., as a component of enzymes involved in photosynthesis (Droux 2004). Poor plant nutrition with sulfur results in a weak nitrogen uptake from the soil. Thus, organic fertilization rich in sulfur seems to be very important. The content of sulfates in the compost mass was decreasing in lignocellulosic compost with chicken feathers after week 14 in compost II and remained constant in compost I (Fig. 4) and at the end of composting amounted to 30 and 150 mg kg^−1^ dw, respectively. In this context, the composts obtained should be considered as valuable in terms of sulfate levels, because sulfate content was very low (< 0.01 mg/kg^−1^) even in mature composts containing chicken manure, as demonstrated by Ksheem et al. (2015).

Sulfate concentrations in water extracts of lignocellulosic composts (PGSF and PSSF) were lower than in the compost mass. Higher sulfate content was recorded in compost I (PGSF) compared to compost II (PSSF). The dynamics of sulfate S changes in compost I water extract was similar to that of the compost mass, while it was different in compost II (Fig. 4). Assuming that sulfates can be an indicator of compost maturity with keratin waste as a composting component (Bohacz and Korniłłowicz-Kowalska 2009b), it was found that this parameter in composts with low feather content, such as compost I (PGSF), could not be taken into consideration, because the level of these compounds decreased during the composting process in compost water extract and solid compost mass. In turn, in composts with a higher feather proportion, such as compost II (PSSF), sulfate content could be considered as a biodegradation index of keratin component and maturity indicator, because the level of this component increased, albeit not continuously.

However, considering fertilizers, the level of sulfates in water extracts of the tested composts seems to be important, as it can support crops with high sulfur requirements, especially from the family *Cruciferae* (*Brassicaceae*) and *Liliaceae*. This is particularly important in the light of reports showing improvements in plant growth parameters under the influence of compost tea spraying (Ezz El-Din and Hendawy 2010). However, there are few literature reports with respect to foliar sulfur fertilization of plants (Tea et al. 2004). These studies underlined the importance of sulfur and sulfate in foliar plant nutrition. Their authors believed that micronutrients bound by sulfates are more easily available, cheaper and less harmful to plants than in the form of other salts. Milinković et al. (2019) reported that both water extracts and compost tea were a source of soluble S, except that soluble S content in compost tea was almost tenfold higher compared to water extracts of green waste composts (leaves and grass). Koné et al. (2010) provided sulfate values in compost tea from chicken manure, sheep manure, bovine manure, and shrimp powder that ranged from 2 to 31 mg/L. Fageria et al. (2009) reported in turn that fertilizer doses of Fe, Mn, Zn, and Cu sulfates used as foliar sprays for arable crops should be within the range of 3–6, 1–2, 1.5–2.5, and 0.5–1 Kg/500 L water, respectively. In Poland, the limits of macro- and micronutrients contained in the soil and foliar fertilizers used in plant cultivation are determined by European Commission Regulation (EC) No 2003/2003 of the European Parliament and the Council of 13 October 2003 on fertilizers (EC Official Journal, L 304, 21.11.2003) as amended (EC Regulation, 2007 of 19 February 2007). According to Szewczuk and Sugier (2009), the content of macronutrients, such as nitrogen and sulfur in multicomponent foliar fertilizers should be 5.0 ≥ 20% of the weight and 1.5 ≥ 5.0% of the weight, respectively. The content of macroelements, including S and total N in water extracts of the studied lignocellulosic composts was presented in the previous work of Bohacz (2018b). It was calculated in lignocellulosic composts I and II (PGSF and PSSF) that the obtained compost water extracts contained less weight percentage of S and N than those described by Szewczuk and Sugier (2009). The percentage of weight of mineral form of nitrogen and sulfur in the compost water extract from week 30 of composting was also low (< 2.0 × 10^−1^). On this basis, it can be assumed that water extracts of both composts cannot serve as sources of easily accessible forms of nitrogen and sulfur in the form of foliar sprays.

Emission of H_2_S was not recorded during the composting process.

### Balance of nitrogen and sulfur in the final compost product

Mineral nitrogen content in the obtained composts was higher in compost I than compost II and ranged from 2.0 to 20.32% and 1.0 to 12% of total N, respectively. The nitrate form (19.0% and 3.40%) predominated over the ammonium form (1% and 3%) in composts I and II, respectively. Similar trends were obtained by Tiquia (2002) in composts containing chicken manure, fodder, and feather residues or pig liquid manure composted with vegetable waste (grass, leaves, bark), and by Wang et al. (2004) during the composting of cattle and pig liquid manure with straw and sawdust. Below 2% of organic N in compost I and 0.5% in compost II entered the water fraction as N-NO_3_^−^ in this study. Calculations carried out in this work showed that the proportion of organic N in the total nitrogen pool of composts was greater in compost II (94%) than compost I (79%).

It was found after 300 weeks of composting that the proportion of sulfate (similar as mineral nitrogen forms) in the total sulfur pool was higher in compost I (5.0–23.0%) than in compost II (1.0–10.0%). Below 1% of organic S in compost I and 0.2% in compost II entered compost water extracts as S-SO_4_^2−^ after 14 weeks.

### Correlation between enzymatic activity and different forms of nitrogen and sulfur in solid fraction

Correlation analysis (*r* Pearson) showed that arylsulfatases were significantly positively responsible for ammonium ion transformations in both composts (Table 1). This suggests that this enzyme plays an important role in organic N mineralization during keratin component composting. However, protease was positively significantly responsible for changes in sulfate ion concentrations in compost I (0.726*) and urease in compost II (− 0.813**). In the literature, there are reports on stimulating or inhibiting sulfate effect on urease activity in the soil environment (Kiss and Simihǎian 2002). However, this depends on the type of soil and sulfate salts (Mg, Ca, K, and Na sulfates). A significant positive correlation between cellulase activity and ammonium ions in compost I (PGSF) and nitrate ions in compost II (PSSF) (Table 1) is an interesting characteristic of the studied composts. Raut et al. (2008) argued that a decrease in C/N could be the cause of this dependence. During composting of lignocellulosic waste the availability of organic carbon decreased and nitrogen availability increased, which favored microbial growth.

**Table 1:** Correlation coefficient (*r*) between microbiological and chemical characteristics in composts containing lignocellulose and keratin waste. The significance level at α=0.05

	Protease	Urease	Cellulase	Arylsulfatase
Compost I				
S-SO_4_^2−^	0.726*	0.648	− 0.034	− 0.587
N-NH_4_^+^	0.015	− 0.339	0.747*	0.983***
N-NO_3_^−^	− 0.872**	− 0.583	− 0.371	0.155
Compost II				
S-SO_4_^2−^	0.123	− 0.813**	0.576	0.373
N-NH_4_^+^	0.198	− 0.643	0.776*	0.744*
N-NO_3_^−^	0.229	− 0.304	0.798**	0.956***

## Conclusion

Composts with different contents of lignocellulosic and keratin waste may be significant for agricultural practice, because both composted mass and compost water extracts contain mineral forms of N and S. Their presence is conditioned by enzymatic activity related to biodegradation and biotransformation of organic matter rich in carbon, nitrogen, and sulfur, i.e., the activity of cellulase, urease, protease and arylsulfatase. The presence of sulfates in the compost mass can be particularly important in terms of fertilization of soils poor in this component. Research results demonstrate that compost with lower amounts of feather and containing more easily available lignocellulosic fraction (especially grass, pine bark, sawdust), may be more important from the fertilizer point of view than compost that contains more hard-to-degrade lignocellulosic waste (wheat straw, sawdust, pine bark) and higher feather content. This is indicated by significantly higher content of mineral nitrogen forms, particularly nitrates in the compost mass and compost water extract. However, the lower level of mineral forms of nitrogen and sulfur in compost water extracts than in the compost mass, in relation to plant requirements, does not predispose these composts as significant fertilizers in the form of foliar sprays.

The application of these types of composts into the soil can support plant cultivation and be important in the context of sustainable agriculture and integrated crop production development.
